# Guidance of development, validation, and evaluation of algorithms for populating health status in observational studies of routinely collected data (DEVELOP-RCD)

**DOI:** 10.1186/s40779-024-00559-y

**Published:** 2024-08-06

**Authors:** Wen Wang, Ying-Hui Jin, Mei Liu, Qiao He, Jia-Yue Xu, Ming-Qi Wang, Guo-Wei Li, Bo Fu, Si-Yu Yan, Kang Zou, Xin Sun

**Affiliations:** 1 Institute of Integrated Traditional Chinese and Western Medicine, Chinese Evidence-Based Medicine and Cochrane China Center, West China Hospital, Sichuan University, Chengdu, 610041 China; 2 NMPA Key Laboratory for Real World Data Research and Evaluation in Hainan, Chengdu, 610041 China; 3 Sichuan Center of Technology Innovation for Real World Data, Chengdu, 610041 China; 4 Center for Evidence-Based and Translational Medicine, Zhongnan Hospital of Wuhan University, Wuhan, 430071 China; 5 Department of Health Research Methods, Evidence and Impact, McMaster University, Hamilton, ON L8S 4L8 Canada; 6 Center for Clinical Epidemiology and Methodology, Guangdong Second Provincial General Hospital, Guangzhou, 510317 China; 7 Biostatistics Unit, Research Institute at St. Joseph’s Healthcare Hamilton, Hamilton, ON L8N 4A6 Canada; 8 School of Computer Science and Engineering, University of Electronic Science and Technology of China, Chengdu, 611731 China; 9 West China School of Public Health and West China Fourth Hospital, Sichuan University, Chengdu, 610041 China

**Keywords:** Routinely collected healthcare data, Algorithms, Health status, Guidance

## Abstract

**Background:**

In recent years, there has been a growing trend in the utilization of observational studies that make use of routinely collected healthcare data (RCD). These studies rely on algorithms to identify specific health conditions (e.g. diabetes or sepsis) for statistical analyses. However, there has been substantial variation in the algorithm development and validation, leading to frequently suboptimal performance and posing a significant threat to the validity of study findings. Unfortunately, these issues are often overlooked.

**Methods:**

We systematically developed guidance for the development, validation, and evaluation of algorithms designed to identify health status (DEVELOP-RCD). Our initial efforts involved conducting both a narrative review and a systematic review of published studies on the concepts and methodological issues related to algorithm development, validation, and evaluation. Subsequently, we conducted an empirical study on an algorithm for identifying sepsis. Based on these findings, we formulated specific workflow and recommendations for algorithm development, validation, and evaluation within the guidance. Finally, the guidance underwent independent review by a panel of 20 external experts who then convened a consensus meeting to finalize it.

**Results:**

A standardized workflow for algorithm development, validation, and evaluation was established. Guided by specific health status considerations, the workflow comprises four integrated steps: assessing an existing algorithm’s suitability for the target health status; developing a new algorithm using recommended methods; validating the algorithm using prescribed performance measures; and evaluating the impact of the algorithm on study results. Additionally, 13 good practice recommendations were formulated with detailed explanations. Furthermore, a practical study on sepsis identification was included to demonstrate the application of this guidance.

**Conclusions:**

The establishment of guidance is intended to aid researchers and clinicians in the appropriate and accurate development and application of algorithms for identifying health status from RCD. This guidance has the potential to enhance the credibility of findings from observational studies involving RCD.

**Supplementary Information:**

The online version contains supplementary material available at 10.1186/s40779-024-00559-y.

## Background

Routinely collected health data (RCD), a byproduct of healthcare systems, are data collected without a prior research purpose. In recent years, RCDs have been increasingly used in observational studies [1–5]. These studies typically rely on algorithms to identify health status [6, 7], which serves as a study variable aligned with the study aim (e.g. study participant, exposure, outcome, or confounders). For example, algorithms may be employed to detect patients presenting with diabetes or sepsis from RCD. These health statuses may be used for selecting a target population, treated as an outcome, or considered a confounding variable in observational studies.

These algorithms may range from simple (e.g., diagnosis codes, operation codes) to highly sophisticated, involving machine learning or deep learning technologies [8, 9]. Regardless of complexity, it is essential that algorithms are well-developed and validated. Their performance should achieve a high level of accuracy to ensure the appropriate identification of the health status of interest with minimal risk of misclassification. This is a crucial prerequisite for observational studies using RCD.

However, there were notable disparities in the development and validation of algorithms in RCD studies. A systematic review revealed a wide variation in the algorithms used to identify rheumatoid arthritis, ranging from a single international classification of diseases (ICD) code to 9 ICD codes accompanied by medications and laboratory data, with positive predictive value (PPV) ranging from 66 to 97% [10]. Consequently, due to these substantial variations, most algorithms used in RCD studies are considered less optimal [11–13]. Many algorithms with low accuracy often lead to misclassification of health status, which can introduce bias into study results [11, 14]. For instance, outcome misclassification may distort relative risk by up to 48% [14].

In recent years, there has been a growing focus on the potential risks associated with misclassifying health status [15–18]. While previous efforts have primarily concentrated on reporting algorithms and their validations [1, 2, 19], there has been limited attention given to systematically guiding the development, validation, and evaluation of algorithms for identifying health status in the context of RCD studies. To address these significant methodological gaps, we have developed guidance to assist in the development, validation, and application of algorithms for identifying health status in RCD studies.

## Methods

We systematically developed guidance for the development, validation, and application of algorithms to identify health status (DEVELOP-RCD).

### Conceptualization and generation of the guidance

We formed a research team comprising experts in clinical epidemiology, biostatistics, and artificial intelligence to conceptualize and develop the initial guidance.

The team commenced by conducting a narrative review through a PubMed search to identify methodology reviews or example studies related to the development and validation of algorithms. The detailed literature search findings are presented in Additional file 1. We synthesized concepts and methods from the included studies, as well as systematically surveyed the validation and impact of algorithms to pinpoint important methodological gaps in observational studies using routinely collected data [20]. Subsequently, we carried out a practical study utilizing an algorithm for sepsis identification as an illustrative example.

Drawing from the narrative review, systematic survey, and empirical example, the research team conceptualized the working process and formulated key methodological items for developing, validating, and evaluating the algorithm.

### Consensus of the guidance documents

We convened a group of 20 external experts to participate in the consensus process. The participants included 8 epidemiologists, 4 statisticians, and 2 information experts from academic institutions. Additionally, we invited 1 journal editor and 5 information experts from data companies.

The external experts were initially tasked with independently reviewing the initial guidance document via email. Specifically, they were asked to assess the completeness, importance, and potential inclusion of the items. Experts were also consulted on whether any additional contexts and items should be considered. Subsequently, we updated the list of recommendation items based on the feedback received from the external experts.

A formal consensus meeting was then held to finalize the guidance. Before the meeting, we provided the updated guidance for preview by external experts. During the meeting, expert opinions on the importance of proposed guidance items were sought. We calculated agreement percentages on item importance among participants. Consensus was defined as a percentage over 80%. Any discrepancies were resolved through discussion among participants and research group members. If necessary, participants were asked to vote on unresolved issues.

## Results

### Literature review

The narrative review comprised 28 reports, including 7 methodology reviews and 21 example studies. The 7 methodology reviews deliberated on the concepts, working process, as well as design and analytical methods of algorithm development and validation, with 6 of them specifically involving machine learning. Detailed information regarding the 28 reports has been presented in Additional file 2: Table S1. Among the 21 examples, 10 utilized machine learning in algorithm development. Results from the systematic survey on validation and impact of algorithms were previously published elsewhere [20]. In brief, our systematic survey identified significant methodological issues in the validation and interpretation of algorithms in observational studies of RCD: only 26.6% of studies used validated algorithms; more than 50% of validation studies may provide biased estimates of sensitivity and specificity; when using alternative algorithms, 18.2–45.5% of studies yield differential effect estimates.

### Guidance development process

Based on the comprehensive literature review, our research group initially developed a methodological workflow comprising 4 consecutive steps for developing, validating, and evaluating an algorithm. Working goals were established for each step through a series of 4 interactive meetings. Building upon this foundational workflow, the research group subsequently formulated a total of 14 practical recommendations covering the 4 critical steps.

The generated guidance, which included the workflow and practical recommendations, was reviewed by external experts who collectively endorsed the proposed workflow while providing suggestions for refining 3 specific recommendation items. Subsequently, the research group revised the descriptions of these 3 items based on their feedback. No additional items were recommended for inclusion or removal.

During the consensus meeting, the generated workflow was re-confirmed. However, one of the practical recommendation items did not achieve a consensus and was deemed unimportant (i.e., blinding of reviewers during review of medical records when using medical chart review as the reference standard). This item was subsequently removed. The wording for each of the remaining recommendation items was further refined.

**Fig. 1:**
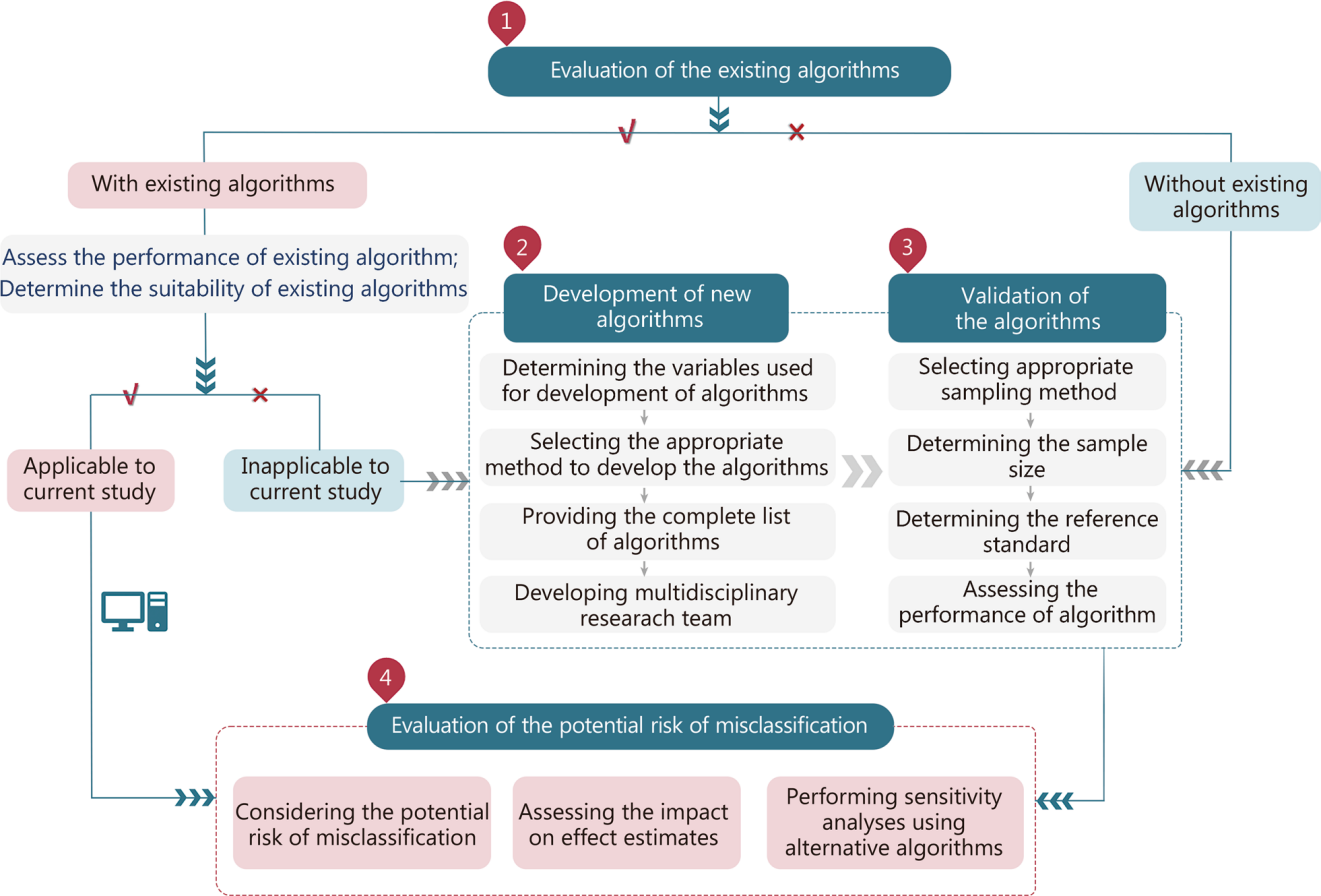
Workflow for development, validation, and evaluation of algorithms for populating health status in observational studies of routinely collected healthcare data (DEVELOP-RCD)

The final guidance (DEVELOP-RCD) consisted of a standardized workflow to facilitate the development, validation, and evaluation of algorithms (Fig. 1), along with specific recommendations for consolidating good practices.

### Standardized workflow

Typically, it is essential to establish a framework for target health status before developing an algorithm. This framework should encompass the setting in which routinely collected data were generated, the medical definition of the health status, and the timing of identifying the health status. For instance, when developing an algorithm to identify sepsis, one would understand the clinical criteria for defining sepsis (e.g., Sepsis-3 criteria), determine the type of data [e.g. electronic medical records, claims data, intensive care units (ICU) registry], and decide whether the sepsis is identified for post-hoc monitoring or supporting real-time diagnosis.

When aiming for a specific health status, one should first search to ascertain the availability of an existing algorithm and evaluate its suitability for the target health status. In numerous instances, algorithms may have already been developed, and reviewing these existing algorithms can assist researchers in determining their appropriateness for the target health status.

The assessment of suitability will be based on the performance of the current algorithm and its alignment with the target health status framework. If the algorithm’s performance is poor or if the setting, timing, or medical definition are inconsistent with the target framework, it may not be deemed suitable. Notably, the assessment of suitability can sometimes be subjective and dependent on how well existing algorithms meet research requirements.

If an existing algorithm is considered unsuitable or if no suitable algorithms are available, a new algorithm must be developed, validated, and evaluated for its impact on the results. The algorithms can range from simple single codes such as diagnosis codes (e.g., International Classification of Diseases Codes) to complex machine learning using multiple variables. It is important to carefully select potential variables for algorithm development and choose appropriate methods (e.g., codes-based, rule-based algorithms, or machine learning methods).

When conducting a validation study, it is crucial to carefully consider methodological aspects such as the approach to population sampling, sample size determination, selection of an appropriate reference standard, and the application of suitable statistical methods for assessing accuracy estimates [e.g., sensitivity, specificity, positive predictive value (PPV), negative predictive value (NPV)]. Furthermore, it is essential to evaluate the potential risk of algorithm misclassification and assess how any resulting bias may impact effect estimation. This can be achieved through correction or quantification of potential misclassification bias and by performing sensitivity analyses to ensure the robustness of study findings (Fig. 1).

### Recommendations for good practice

The standardized workflow served as the basis for developing specific recommendations aimed at consolidating good practice. Additionally, a set of elaborations was developed to facilitate the implementation of the guidance.

#### Assess the availability of algorithms related to the targeted health status

Researchers should evaluate the presence of pre-existing algorithms through a thorough exploration of published articles, websites, or databases. While these existing algorithms may not always be directly applicable, gaining insight into them can greatly assist in refining algorithms for future studies [21].

#### Upon the development of an algorithm, it is essential to assess whether the existing algorithms are applicable to the current study

The judgment involves an evaluation of the performance of existing algorithms and an assessment of the suitability of the database.

*Assess whether the performance of existing algorithms is sufficient for application to the current research question.* The evaluation should take into account the type of health status (e.g., participants, exposure, and outcome), the feature of health status (e.g., mild or severity, easy or difficult to diagnose, ignored or recognized in the population), and database profile (e.g., how medical codes were recorded in the healthcare system) for identification or classification purposes. The impact of algorithm accuracy on effect estimates may vary under different scenarios. For instance, when classifying exposure, high sensitivity is generally more important than specificity and PPV. Conversely, high specificity is crucial for classifying outcomes. Moreover, a high PPV is essential for identifying study participants, while a high NPV is important for excluding participants who meet exclusion criteria [14]. The assessment of accuracy necessitates access to validation information and a comprehensive inventory of algorithms. In the absence of essential information, evaluations may become unfeasible or inaccurate, prompting investigators to develop new algorithms.

*Determine the suitability of existing algorithms for the database utilized in the present study.* The participant’s characteristics, coding systems, time frames, and data resources exhibit notable variations across data sources [22, 23]. The performance of algorithms may also demonstrate significant differences between data sources [10, 19, 24]. Therefore, researchers should potentially impact of these differences on algorithm performance. Such assessments necessitate a comprehensive understanding of the database, including the consistency of coding systems, time frames, and participant characteristics across databases, as well as the clinical granularity of the data source.

#### Identify the essential data elements required for the development of algorithms and evaluate the accuracy and comprehensiveness of these data elements

Before developing the algorithms, researchers should carefully consider the inclusion of specific data elements (e.g. diagnosis code, lab results, prescription record) into the algorithms [21]. It is imperative to establish a fundamental set of data elements and assess whether these essential components have been accurately and comprehensively captured in the database for algorithm development. Accuracy pertains to the ability of data elements to faithfully represent a patient’s clinical condition as documented in routine care, while completeness refers to the comprehensive documentation of all necessary data elements in the database.

#### Choose a suitable approach for developing a new algorithm

The characterization of health status involves a spectrum from simplicity to complexity based on the number of clinical factors involved and whether it is objective or subjective [6, 25]. Researchers are advised to choose suitable methodologies when developing new algorithms based on the specific nature of the targeted health status. Generally speaking, approaches for developing novel algorithms include codes (i.e., diagnosis codes, operation codes), rule-based models (i.e., combinations of laboratory findings and diagnosis codes), and machine learning techniques [2, 6, 26, 27]. For straightforward and objective health statuses, researchers might consider employing codes or rule-based models [28–31], while in cases involving intricate or subjective study variables, machine learning methods could be preferable [32, 33].

#### Detailed reporting of the algorithms, either in the manuscript or included as an appendix

It is essential to explicitly report the algorithms to ensure the transparency of study findings and the reproducibility of research. To facilitate research reproducibility, researchers should provide a comprehensive list of algorithms either in the manuscript or as an appendix [1, 2]. This detailed list should include, among other things, information regarding the criteria employed for defining health status and the codes or data elements used in algorithm development.

#### Develop a cross-disciplinary research team

The development of algorithms requires a diverse range of expertise [8, 21]. A research team comprising experts from various disciplines is essential to ensure the scientific rigor of the algorithms. Typically, this includes participation from clinicians, epidemiologists, and informationists. Clinicians contribute relevant background knowledge related to the hypotheses under consideration, epidemiologists assist in selecting appropriate methods for developing algorithms, and informationists provide comprehensive insights into database profiles. In cases involving machine learning, it is important to involve artificial intelligence specialists.

#### Choose a suitable sampling method to guarantee representation in the selected cases

An appropriate sampling method should ensure that the selected population is accurately represented. If an inappropriate sampling method is used, the characteristics and prevalence of health-status-positive individuals in the sampled population may differ from those in the target population, leading to biased estimates [18]. Therefore, researchers should carefully consider the sampling method employed. Generally, sampling methods include cross-sectional sampling, case–control sampling, and test-results-based sampling. Cross-sectional sampling involves randomly selecting the study population from a database; case–control sampling entails random selection from the health-status-positive and health-status-negative populations based on a gold/reference standard; while test-results-based sampling refers to random selection from algorithm-positive and algorithm-negative populations [34, 35]. Due to changes in prevalence, case–control sampling often biases the estimates of PPV and NPV; similarly, test results-based sampling can bias sensitivity and specificity estimates [18, 35, 36]. An inappropriate sampling method may lead to bias in any direction.

#### Ensure that the sample size is sufficiently large to achieve the desired level of precision in accuracy estimation

To ensure adequate sample size, researchers should estimate the minimum sample size required for validation studies by specifying the desired precision for the accuracy estimates. The sample size can be calculated based on the desired width of the 95% confidence interval (CI) for anticipated accuracy estimates [37, 38].

#### Select a gold standard or reference standard to classify individuals with or without a given health status

The validity of algorithms should be estimated against a gold standard or reference standard. However, a true gold standard is rarely available, so validation studies often rely on reference standards to classify the health or exposure status of individuals [14, 39]. Using an imperfect reference standard may lead to biased estimates of algorithm accuracy [36, 40], emphasizing the importance of carefully selecting the reference standard based on its availability, accuracy, and completeness [21, 41]. For instance, a medical chart review is commonly used as a reference standard [42]. When using medical chart review as a reference standard, researchers should consider whether the chart abstraction includes all essential information related to the classification of health or exposure status, whether the information is accurately recorded, and whether it is complete for both look-back and look-forward periods relative to the date of potential health or exposure status [42, 43].

#### Assess the performance of the algorithms

*Calculate and report at least four estimates of accuracy.* Similar to a diagnostic test, the standard parameters for accuracy assessment include sensitivity, specificity, PPV, and NPV [19]. To enhance comprehension of algorithm performance and their appropriate application in further research, it is recommended that researchers calculate and report these 4 performance measures at a minimum. Furthermore, 95% CI should be calculated and reported for each of these measures [19].

*If using case–control sampling or test results-based sampling, researchers should employ an appropriate approach to mitigate any potential bias.* As mentioned previously, the use of case–control sampling and test results-based sampling often introduces bias into accuracy estimates [18, 34, 35]. When employing these methods, researchers should address the validity of the estimates and consider using appropriate techniques to mitigate any potential bias [34]. For instance, in a study that utilized test results-based sampling to assess algorithm performance, corrective measures were taken by extrapolating proportions from both the test-positive and test-negative groups to represent the entire hospitalized population. Subsequently, 10,000 bootstrapped samples were generated to calculate percentile-based CI for accuracy estimates [11].

#### Consider the transportability of the algorithms to different data sources

The performance of algorithms may vary across different data sources. Researchers should consider the generalizability of the algorithms to alternative data settings. If applicable, one should consider conducting an external validation study to evaluate the transportability of the algorithms in a new setting, such as different healthcare institutions and various types of databases (i.e., real-time data) [21]. In general, testing an algorithm in real-time data may help researchers gain a better understanding of its advantages and limitations [44].

#### Consider the potential implications of algorithm misclassification on the study findings

The potential impact of misclassifying health status on study estimates could be significant [14]. Researchers should consider the potential misclassification risk introduced by imperfect algorithms, and evaluate the types of misclassification risk involved. Typically, misclassification bias includes non-differential and differential misclassification [45]. Non-differential misclassification refers to classifying exposure (or disease) as unrelated to disease (or exposure), while differential misclassification involves classifying exposure (or disease) as related to disease (or exposure) [46]. The effects of non-differential and differential misclassification on estimates may differ. Non-differential misclassification in exposures and outcomes tends to bias treatment effect estimates towards the null (no effect) hypothesis, whereas differential misclassification leads to bias in any direction [47, 48].

#### Assess the impact on study estimates

*Use statistical methods to correct or quantify the potential bias arising from misclassification.* To assess the impact of algorithm misclassification on research findings, we suggest employing statistical methods to correct or quantify the potential misclassification bias introduced by imperfect algorithms. Common approaches for addressing misclassification bias include likelihood-based methods [49, 50], such as the Prior Knowledge Guided Integrated Likelihood Estimation method and augmented estimation procedure [51, 52].

In addition, researchers can use quantitative bias analysis to evaluate the impact of misclassification on study estimates [46]. Instead of correcting or reducing the risk of misclassification bias, quantitative bias analysis aims to assess the direction and magnitude of misclassification bias [53, 54]. Recently, several quantitative bias approaches have been developed, such as probabilistic bias analysis and Monte Carlo simulation methods [55, 56].

*Perform sensitivity analyses using alternative algorithms to evaluate the robustness of study findings.* Variations in algorithmic approaches often lead to significant disparities in estimations [11, 57]. These differences are deemed noteworthy when there is an inconsistency between estimations from primary and sensitivity analyses or when a 95% CI for estimation ratios fails to encompass unity. Estimation ratios are derived by dividing those obtained through primary analyses by those from sensitivity analyses. In our previous investigation involving 222 RCD studies, we found that using alternate algorithms for identifying health status resulted in differential effect estimations ranging from 16.7% to 35.7%. Employing alternate algorithms and comparing outcomes can enhance scrutiny regarding result robustness [58]. Such alternate approaches encompass diverse code lists as well as varied variables or methodologies employed for algorithm development.

#### Discuss the potential bias arising from algorithm misclassification

To ensure transparency regarding the risk of bias, researchers should address potential misclassification bias in the discussion section. This includes specifying the type of misclassification bias (differential *vs*. non-differential) and discussing its potential impact on effect estimates (both direction and magnitude of potential bias) [1, 2]. If results vary with alternative algorithms or if their interpretation changes based on quantitative bias analysis, researchers should transparently report these results and interpret them cautiously.

## Practical example—developing an algorithm for sepsis identification

In our previous study, we investigated the frequency of sepsis in ICU-admitted patients using a registry of healthcare-associated infection (HAI) in ICU in West China. The ICU-HAI registry included all patients admitted to ICU at West China Hospital since 2012 and contained detailed information regarding the demographics, vital signs, laboratory results, notes, treatment, and outcomes [59, 60]. To accomplish this objective, an algorithm for identifying sepsis was employed.

### Assessing existing algorithm

To identify patients with sepsis within the ICU-HAI registry, we conducted an extensive review of previous studies focusing on existing algorithms for sepsis. Subsequently, an adult sepsis event (ASE) algorithm tailored for application with electronic healthcare records (EHR) across diverse US hospital settings was developed [11], demonstrating validation with notable metrics including 69.7% sensitivity, and 98.1% specificity along with a PPV of 70.4%, and NPV of 98.0%. Despite its success within US contexts, this algorithm is not suitable for application with Chinese EHR data due to significant differences in patient characteristics, treatment protocols such as blood culture utilization and antimicrobial practices as well as variations between EHR systems utilized in both countries’ healthcare settings. The implementation of this algorithm revealed a mere incidence rate of sepsis at only 4%, derived from analysis within our ICU-HAI registry—significantly lower than anticipated estimates standing at approximately 36.31% [61]. Through comprehensive evaluation, it became evident that these existing algorithms are unsuitable for application within China’s ICU-HAI registry.

### Development of a new algorithm

We developed a new algorithm for identifying sepsis patients based on the ICU-HAI registry. In clinical practice, the diagnostic criteria are complex and involve numerous clinical factors, including the diagnosis of infection, vital signs, lab results, microbiological samples, antimicrobial usage, and vasopressor medications. Therefore, we opted for machine learning methods to handle large numbers of variables and detect the intricate interrelationships among these variables. Our multidisciplinary research team consists of 3 experts in epidemiology, 2 experts in clinical medicine, 2 experts in statistics, and 1 expert in artificial intelligence. Given the complex features of the data involved in this study, we chose the gate recurrent unit-ordinary differential equation-Bayes (GRU-ODE-Bayes) method to deal with time-series data and hundreds of features.

### Validation of the algorithm

In order to evaluate its accuracy in identifying sepsis cases, a validation study was conducted. Given the relatively low prevalence of sepsis, an algorithm-based sampling approach was chosen to ensure an adequate number of positive samples. Specifically, 100 cases and 150 cases were randomly sampled from sepsis-positive patients and sepsis-negative patients, respectively. The reference standard for this assessment was a medical records review, including demographics, vital signs, laboratory results, and treatment details extracted from the records. Four clinicians independently reviewed the medical records abstraction, discussing any discrepancies among themselves. The performance of GRU-ODE-Bayes algorithms and two rule-based algorithms (ASE algorithm and ICD codes) was assessed. To address potential bias in sensitivity and specificity estimates resulting from test result-based sampling, adjustments were made by extrapolating proportions back to the entire study population using bootstrapped samples to calculate CI for the estimates. Through validation, it was found that the GRU-ODE-Bayes algorithm exhibited 81.0% sensitivity (95% CI 74.5–88.3%), 80.5% specificity (95% CI 76.5–85.0%), 60.3% PPV (95% CI 53.6–67.3%), and 92.1% NPV (95% CI 89.2–95.4%). In contrast, the rule-based algorithms demonstrated low sensitivity (ICD codes: 39.9%, 95% CI 35.2–46.0%; ASE algorithm: 5.6%, 95% CI 3.6–7.7%).

### Evaluating the algorithm

Using ICD codes, the ASE algorithm, and the GRU-ODE-Bayes algorithm, we identified 2646, 642, and 8164 patients with sepsis, respectively. The incidence of sepsis among ICU patients was 11.7%, 2.8%, and 36.2% according to the ICD codes, ASE algorithm, and GRU-ODE-Bayes algorithm, respectively. We used the Rogan-Gladen formula for quantitative bias analysis of prevalence to estimate the adjusted incidence, and the estimated incidence of sepsis was found to be 27.1% [62]. Based on this adjusted incidence estimation, it was determined that the GRU-ODE-Bayes algorithm overestimated sepsis incidence by 33.5%, while the ICD algorithm led to an underestimation of incidence by 55.0%.

## Discussion

The potential compromise of the study findings’ validity due to the misclassification of health status by imperfect algorithms is a critical concern [63–66]. It is imperative to employ algorithms with high accuracy and minimal misclassification to ensure the reliability of results [1, 2, 67]. Our previous systematic literature review revealed that only 26.6% of studies used validated algorithms for identifying health status [20]. Even when validated, the methodological quality and performance of validation were often suboptimal, with researchers frequently overlooking their impacts on the results [20].

To improve algorithm transparency and usability, we have systematically developed guidance (DEVELOP-RCD). This comprehensive framework includes a 4-step workflow designed to facilitate sequential progress in algorithm development, validation, and evaluation. Additionally, we have formulated 13 good practice recommendations corresponding to each of these steps.

The guidance has wide-ranging applicability across studies reliant on routinely collected data, such as observation studies or pragmatic trials utilizing RCD. We expect this resource will significantly aid researchers and other users in accurately developing and applying algorithms within RCD-based studies.

## Strengths and limitations

Our study possesses several strengths. Firstly, this guidance comprehensively addresses methodological issues related to the development, validation, and application of algorithms for identifying health status in studies using RCD. Secondly, this guidance was formulated using a rigorous approach. We conducted a narrative review, and a comprehensive survey, and provided empirical examples as part of conceptualizing its initial guidance. Additionally, 20 experts were invited to review the guidance, and their feedback was incorporated into finalizing them. Thirdly, the findings are structured systematically, hence beneficial throughout all stages of algorithm development, validation, and application for identifying health status.

Nonetheless, this study does have certain limitations. Firstly, despite developing this guidance through a systematic approach and consulting extensively during its development phase, additional useful items may emerge in the future. Secondly, the proposed approaches lack widespread validation across diverse study settings. However, this is planned as part of our upcoming steps. Thirdly, due to rapid advancements in information technology, the methods utilized for algorithmic development are evolving swiftly. Hence, it is anticipated that periodic updates will be made to encompass emerging developments.

## Conclusions

Misclassification of health status resulting from imperfect algorithms may pose a serious threat to the validity of study findings, and addressing this issue involves complex methodological considerations. The guidance systematically addresses issues related to the development, validation, and evaluation of algorithms. Ultimately, improved algorithms would enhance the credibility of study findings.

### Supplementary Information

**Additional file 1:** Search strategy.

**Additional file 2:** **Table S1** Overview of the 28 articles included in the narrative review.

## Data Availability

Not applicable.

## Abbreviations

ASE: Adult sepsis event
CI: Confidence interval
DEVELOP-RCD: Development, validation, and evaluation of algorithms for populating health status in observational studies of routinely collected data
EHR: Electronic healthcare records
GRU-ODE-Bayes: Gate recurrent unit-ordinary differential equation-Bayes
HAI: Healthcare-associated infection
ICD: International classification of diseases
ICU: Intensive care units
NPV: Negative predictive value
PPV: Positive predictive value
RCD: Routinely collected healthcare data
