# Supplementary material for: Guidance of development, validation, and evaluation of algorithms for populating health status in observational studies of routinely collected data (DEVELOP-RCD)
Source: Mil Med Res. 2024 Aug 6;11:52. doi: 10.1186/s40779-024-00559-y (PMC11302358; doi:10.1186/s40779-024-00559-y)
Supplement: Supplementary file 1 — Additional file 1: Search strategy. [file 40779_2024_559_MOESM1_ESM.pdf]

Search strategy

#1. algorithm*[tiab] OR algorithm*[tw] OR algorithm*[MeSH Major Topic]

#2. (((“machine learning” [tiab] OR “machine learning” [tw] OR “machine learning”[MeSH Major Topic]) OR (“artificial intelligence”[tiab] OR “artificial intelligence”[tw] OR “artificial intelligence” [MeSH Major Topic])) OR (“deep learning”[tiab] OR “deep learning”[tw] OR “deep learning”[MeSH Major Topic]))

#3. #1 OR #2

#4. “administrative” [tiab] OR “claims” [tiab] OR “routine data” [tiab] OR “routinely collected” [tiab] OR "retrospective database"[tiab] OR "secondary data"[tiab] OR “medical insurance” [tiab] OR “electronic health record” [tiab] OR “electronic healthcare record” [tiab] OR “EHR” [tiab] OR “electronic medical record” [tiab] OR “EMR” [tiab]

#5. #3 AND #4

#6. ((Address[ptyp] OR Biography[ptyp] OR Bibliography[ptyp] OR Autobiography[ptyp] OR Case Reports[ptyp] OR Clinical Conference[ptyp] OR Comment[ptyp] OR Congress[ptyp] OR Consensus Development Conference[ptyp] OR Consensus Development Conference, NIH[ptyp] OR Editorial[ptyp] OR Letter[ptyp] OR Dictionary[ptyp] OR Directory[ptyp] OR Historical Article[ptyp] OR Legal Case[ptyp] OR Meta-Analysis[ptyp] OR Guideline[ptyp] OR News[ptyp] OR Newspaper Article[ptyp] OR Patient Education Handout[ptyp] OR Personal Narrative[ptyp] OR Interview[ptyp] OR Legislation[ptyp] OR Lecture[ptyp] OR Video-Audio Media[ptyp] OR Webcasts[ptyp] OR Portrait[ptyp]))

#7. #5 NOT #6

#8. Filters: in the last 10 years, humans English

#9. Search date: January 15th, 2021
