# Supplementary material for: Guidance of development, validation, and evaluation of algorithms for populating health status in observational studies of routinely collected data (DEVELOP-RCD)
Source: Mil Med Res. 2024 Aug 6;11:52. doi: 10.1186/s40779-024-00559-y (PMC11302358; doi:10.1186/s40779-024-00559-y)
Supplement: Supplementary file 2 — Additional file 2: Table S1 Overview of the 28 articles included in the narrative review. [file 40779_2024_559_MOESM2_ESM.pdf]

Table S1 Overview of the 28 articles included in the narrative review

Author	Title	Summary of the study	Significance of the current study
Methodology reviews (*n* = 7)			
Banda et al. [1] (2018)	Advances in electronic phenotyping: from rule-based definitions to machine learning models	This study reviewed methodologies for algorithm development, encompassing both rule-based and machine learning approaches	How to evaluate the existing algorithms and develop a new algorithm
Basile et al. [2] (2018)	Informatics and machine learning to define the phenotype	This review discussed algorithm development methodologies for classifying health status. Specifically, the authors highlight promising machine learning approaches for understanding complex traits and classifying patients	How to develop a new algorithm using machine learning
Doupe et al. [3] (2019)	Machine learning for health services researchers	This review presented a process for the development of machine learning algorithms, including methodologies for selecting appropriate models	How to develop a new algorithm using machine learning
Jamshidi et al. [4] (2019)	Machine learning-based patient-specific prediction models for knee osteoarthritis	This study reviewed and discussed methods for identifying and classifying patients with osteoarthritis, especially for machine learning-based approaches	How to develop new algorithms, especially for using machine learning-based approaches.
Richesson et al. [5] (2016)	Clinical phenotyping in selected national networks: demonstrating the need for high-throughput, portable, and computational methods	This review outlined the advantages and disadvantages of both rule-based and machine learning methods for algorithm development	How to select the appropriate method for algorithm development.
Wong et al. [6] (2018)	Using machine learning to identify health outcomes from electronic health record data	This review illustrated four scenarios of when and how to use machine learning to identify health outcomes from electronic healthcare data	How to develop a new algorithm using machine learning
Mo et al. [7] (2015)	Desiderata for computable representations of electronic health records-driven phenotype algorithms	This study proposed 10 desired characteristics for developing a computable phenotype representation model using electronic healthcare data	How to evaluate the existing algorithms and develop a new algorithm

Author	Title	Summary of the study	Significance of the current study
Example studies (*n* = 21)			
Culpepper et al. [8] (2019)	Validation of an algorithm for identifying MS cases in administrative health claims datasets	This study validated several rule-based algorithms for identifying multiple sclerosis cases in administrative health claims datasets	Example studies for validating the algorithms
Salemi et al. [9] (2018)	Identifying algorithms to improve the accuracy of unverified diagnosis codes for birth defects	This study developed several rule-based algorithms to identify cases with birth defects and found that the completeness of data may impact the accuracy of case finding	Example studies for developing the algorithms
Omino et al. [10] (2017)	The validity of HCC diagnosis codes in chronic hepatitis B patients in the Veterans Health Administration	This study compared the accuracy of different rule-based algorithms for identifying hepatocellular carcinoma cases using administrative databases, finding that using appropriate codes from outpatient or inpatients can accurately identify cases	Example studies for validating the algorithms
Youngson et al. [11] (2016)	Defining and validating comorbidities and procedures in ICD-10 health data in ST-elevation myocardial infarction patients	This study validated the ICD-10 code, procedure codes for ST-elevation myocardial infarction, coronary artery bypass grafting, and percutaneous coronary intervention. The result showed that ICD-10 and the Canadian Classification of Health Interventions code can accurately identify patients with STEMI and define cardiac procedures	Example studies for validating the algorithms
Khurshid et al. [12] (2016)	A simple and portable algorithm for identifying atrial fibrillation in the electronic medical record	In this study, authors developed and validated seven algorithms using rule-based methods and compared them with published ones, the results showed that well-developed rule-based algorithms can provide optimal power for EMR-based atrial fibrillation identification	Example studies for developing and validating the algorithms

Author	Title	Summary of the study	Significance of the current study
Gladstone et al. [13] (2016)	Sensitivity and specificity of administrative mortality data for identifying prescription opioid-related deaths	This empirical study substantiated that prescription opioid-related deaths can be well identified using rule-based algorithms., however, the accuracy of algorithms varied when using different data elements	Example studies for developing and validating the algorithms
Navar-Boggan et al. [14] (2015)	Accuracy and validation of an automated electronic algorithm to identify patients with atrial fibrillation at risk for stroke	This study developed and validated rule-based algorithms for identifying patients with atrial fibrillation, and the result showed that the quality of data may impact the accuracy of algorithms	Example studies for developing and validating the algorithms
McIsaac et al. [15] (2015)	Identifying obstructive sleep apnea in administrative data: a study of diagnostic accuracy	This study developed and validated rule-based algorithms for identifying patients with obstructive sleep apnea, and the results showed that the combination of several data elements may help to improve the PPV of algorithms	Example studies for developing rule-based algorithms
Ducharme et al. [16] (2013)	Validation of diagnostic codes for intussusception and quantification of childhood intussusception incidence in Ontario, Canada: a population-based study	In this study, authors validated an algorithm based on ICD-9 and ICD-10 codes and found the accuracy of algorithms was time-varying	Example studies for validating the algorithms
Castro et al. [17] (2015)	Validation of electronic health record phenotyping of bipolar disorder cases and controls	In this study, the authors employed a range of rule-based algorithms, each constructed using distinct sets of codes, and found that validity varied between different algorithms	Example studies for validating the algorithms
Johnson et al. [18] (2018)	A comparative analysis of sepsis identification methods in an electronic database	This study compared five different algorithms for identifying patients with sepsis. Important divergences in the identification of sepsis were found when using the different algorithms	Example studies for validating and applying the algorithms

Author	Title	Summary of the study	Significance of the current study
Giannini et al. [19] (2019)	A machine learning algorithm to predict severe sepsis and septic shock: development, implementation, and impact on clinical practice	In this study, authors developed a machine-learning algorithm to identify patients with sepsis	Example studies for developing machine-learning algorithm
Corey et al. [20] (2016)	Development and validation of an algorithm to identify nonalcoholic fatty liver disease in the electronic medical record	In this study, the authors utilized models to automatically select features and develop identification algorithms, discovering that machine learning methods outperformed ICD codes alone	Example studies for developing and validating the algorithms
Chan et al. [21] (2016)	Application of recursive partitioning to derive and validate a claims-based algorithm for identifying keratinocyte carcinoma (nonmelanoma skin cancer)	In this study, the authors validated an algorithm for identifying keratinocyte carcinoma and found the algorithm performed well when using an independent data set from a hospital clinic	Example studies for validating the algorithms
Harrison et al. [22] (2015)	Developing the surveillance algorithm for detection of failure to recognize and treat severe sepsis	In this study, a machine-learning algorithm was developed to identify patients with sepsis	Example studies for developing machine-learning algorithm
Bronsert et al. [23] (2020)	Identification of postoperative complications using electronic health record data and machine learning	In this study, authors developed a machine-learning algorithm to identify patients with postoperative complications	Example studies for developing machine-learning algorithm
Seymour et al. [24] (2019)	Derivation, validation, and potential treatment implications of novel clinical phenotypes for sepsis	In this study, authors developed a machine-learning algorithm to identify patients with sepsis	Example studies for developing machine-learning algorithm
Delahanty et al. [25] (2019)	Development and evaluation of a machine learning model for the early identification of patients at risk for sepsis	In this study, the authors developed a machine-learning algorithm to identify sepsis patients and compared its performance to that of a rule-based algorithm. They found that the machine-learning approach exhibited a higher level of sensitivity and precision	Example studies for developing and validating the algorithms

Author	Title	Summary of the study	Significance of the current study
Nielsen et al. [26] (2017)	A predictive model to identify Parkinson's disease from administrative claims data	In this study, the authors developed a machine-learning algorithm capable of identifying Parkinson's disease from administrative claims data	Example studies for developing machine-learning algorithm
Weissman et al. [27] (2017)	Validation of an administrative definition of ICU admission using revenue center codes	In this study, the authors reported that their developed machine-learning algorithm facilitated the precise identification of ICU admissions within administrative databases	Example studies for validating the algorithms
Somnay et al. [28] (2017)	Improving diagnostic recognition of primary hyperparathyroidism with machine learning	In this study, a machine learning algorithm for classifying patients with primary hyperparathyroidism was developed and validated	Example studies for developing and validating the machine-learning algorithms

ICD international classification of diseases, *MS* multiple sclerosis, *HCC* hepatocellular carcinoma, *STEMI* ST-elevation myocardial infarction, *EMR* electronic medical records, *ICU* intensive care unit

References

1. Banda JM, Seneviratne M, Hernandez-Boussard T, Shah NH. Advances in electronic phenotyping: from rule-based definitions to machine learning models. Annu Rev Biomed Data Sci. 2018;1:153-68.
2. Basile AO, Ritchie MD. Informatics and machine learning to define the phenotype. Expert Rev Mol Diagn. 2018;18(3):219-26.
3. Doupe P, Faghmous J, Basu S. Machine learning for health services researchers. Value Health. 2019;22(7):808-15.
4. Jamshidi A, Pelletier JP, Martel-Pelletier J. Machine-learning-based patient-specific prediction models for knee osteoarthritis. Nat Rev Rheumatol. 2019;15(1):49-60.
5. Richesson RL, Sun J, Pathak J, Kho AN, Denny JC. Clinical phenotyping in selected national networks: demonstrating the need for high-throughput, portable, and computational methods. Artif Intell Med. 2016;71:57-61.
6. Wong J, Horwitz MM, Zhou L, Toh S. Using machine learning to identify health outcomes from electronic health record data. Curr Epidemiol Rep. 2018;5(4):331-42.
7. Mo H, Thompson WK, Rasmussen LV, Pacheco JA, Jiang G, Kiefer R, et al. Desiderata for computable representations of electronic health records-driven phenotype algorithms. J Am Med Inform Assoc. 2015;22(6):1220-30.
8. Culpepper WJ, Marrie RA, Langer-Gould A, Wallin MT, Campbell JD, Nelson LM, et al. Validation of an algorithm for identifying MS cases in administrative health claims datasets. Neurology. 2019;92(10):e1016-28.
9. Salemi JL, Rutkowski RE, Tanner JP, Matas JL, Kirby RS. Identifying algorithms to improve the accuracy of unverified diagnosis codes for birth defects. Public Health Rep. 2018;133(3):303-10.
10. Omino R, Mittal S, Kramer JR, Chayanupatkul M, Richardson P, Kanwal F. The validity of HCC diagnosis codes in chronic hepatitis B patients in the veterans health administration. Dig Dis Sci. 2017;62(5):1180-5.
11. Youngson E, Welsh RC, Kaul P, McAlister F, Quan H, Bakal J. Defining and validating comorbidities and procedures in ICD-10 health data in ST-elevation myocardial infarction patients. Medicine (Baltimore). 2016;95(32):e4554.
12. Khurshid S, Keaney J, Ellinor PT, Lubitz SA. A simple and portable algorithm for identifying atrial fibrillation in the electronic medical record. Am J Cardiol. 2016;117(2):221-5.
13. Gladstone E, Smolina K, Morgan SG, Fernandes KA, Martins D, Gomes T. Sensitivity and specificity of

administrative mortality data for identifying prescription opioid-related deaths. CMAJ. 2016;188(4):E67-E72.

14. Navar-Boggan AM, Rymer JA, Piccini JP, Shatila W, Ring L, Stafford JA, et al. Accuracy and validation of an automated electronic algorithm to identify patients with atrial fibrillation at risk for stroke. Am Heart J. 2015;169(1):39-44 e2.
15. McIsaac DI, Gershon A, Wijeysundera D, Bryson GL, Badner N, van Walraven C. Identifying obstructive sleep apnea in administrative data: a study of diagnostic accuracy. Anesthesiology. 2015;123(2):253-63.
16. Ducharme R, Benchimol EI, Deeks SL, Hawken S, Fergusson DA, Wilson K. Validation of diagnostic codes for intussusception and quantification of childhood intussusception incidence in Ontario, Canada: a population-based study. J Pediatr. 2013;163(4):1073-9 e3.
17. Castro VM, Minnier J, Murphy SN, Kohane I, Churchill SE, Gainer V, et al. Validation of electronic health record phenotyping of bipolar disorder cases and controls. Am J Psychiatry. 2015;172(4):363-72.
18. Johnson AEW, Aboab J, Raffa JD, Pollard TJ, Deliberato RO, Celi LA, et al. A comparative analysis of sepsis identification methods in an electronic database. Crit Care Med. 2018;46(4):494-9.
19. Giannini HM, Ginestra JC, Chivers C, Draugelis M, Hanish A, Schweickert WD, et al. A machine learning algorithm to predict severe sepsis and septic shock: development, implementation, and impact on clinical practice. Crit Care Med. 2019;47(11):1485-92.
20. Corey KE, Kartoun U, Zheng H, Shaw SY. Development and validation of an algorithm to identify nonalcoholic fatty liver disease in the electronic medical record. Dig Dis Sci. 2016;61(3):913-9.
21. Chan AW, Fung K, Tran JM, Kitchen J, Austin PC, Weinstock MA, et al. Application of recursive partitioning to derive and validate a claims-based algorithm for identifying keratinocyte carcinoma (nonmelanoma skin cancer). JAMA Dermatol. 2016;152(10):1122-7.
22. Harrison AM, Thongprayoon C, Kashyap R, Chute CG, Gajic O, Pickering BW, et al. Developing the surveillance algorithm for detection of failure to recognize and treat severe sepsis. Mayo Clin Proc. 2015;90(2):166-75.
23. Bronsert M, Singh AB, Henderson WG, Hammermeister K, Meguid RA, Colborn KL. Identification of postoperative complications using electronic health record data and machine learning. Am J Surg. 2020;220(1):114-9.
24. Seymour CW, Kennedy JN, Wang S, Chang CH, Elliott CF, Xu Z, et al. Derivation, validation, and potential treatment implications of novel clinical phenotypes for sepsis. JAMA. 2019;321(20):2003-17.
25. Delahanty RJ, Alvarez J, Flynn LM, Sherwin RL, Jones SS. Development and evaluation of a machine

learning model for the early identification of patients at risk for sepsis. Ann Emerg Med. 2019;73(4):334-44.

26. Searles Nielsen S, Warden MN, Camacho-Soto A, Willis AW, Wright BA, Racette BA. A predictive model to identify parkinson disease from administrative claims data. Neurology. 2017;89(14):1448-56.
27. Weissman GE, Hubbard RA, Kohn R, Anesi GL, Manaker S, Kerlin MP, et al. Validation of an administrative definition of ICU admission using revenue center codes. Crit Care Med. 2017;45(8):e758-62.
28. Somnay YR, Craven M, McCoy KL, Carty SE, Wang TS, Greenberg CC, et al. Improving diagnostic recognition of primary hyperparathyroidism with machine learning. Surgery. 2017;161(4):1113-21.
